# The Importance of Venous Reflux Status Evaluation in the Intensive Care Unit

**DOI:** 10.14336/AD.2023.0731

**Published:** 2024-05-07

**Authors:** Hua Zhao, Beijun Gao, Hongmin Zhang, Xiaoting Wang, Critical Care Ultrasound Study Group

**Affiliations:** Department of Critical Care Medicine, Peking Union Medical College Hospital, Peking Union Medical College, Chinese Academy of Medical Sciences, Beijing 100730, China

**Keywords:** ultrasonography, venous reflux status, hemodynamic therapy, critically ill

## Abstract

The current management approach for critically ill patients emphasizes maintaining adequate cardiac output and mean arterial pressure. Recently, researchers have increasingly emphasized the clinical significance of venous reflux. Bedside venous Doppler ultrasonography offers continuous, dynamic, and quantifiable assessment of the venous reflux status. In this review, we explore the pertinent literature on assessing the venous reflux status in critically ill patients. We propose a bedside ultrasonographic evaluation method that starts with the hepatic veins and progresses to the portal, renal and intrarenal, femoral, and pulmonary veins. The clinical significance of venous reflux status evaluation is discussed in terms of its effect on right ventricular function, the functioning of other organs, and the guidance of fluid therapy. Overall, we underscore the importance of venous reflux status evaluation in critically ill patients and highlight the benefits of incorporating bedside ultrasonography for the continuous monitoring of venous return.

Traditionally, the hemodynamic management of critically ill patients focuses on the arterial supply side to ensure organ perfusion [1].﻿ Current guidelines endorse fluid resuscitation and administration of vasoactive drugs, which aim to increase arterial circulatory flow [1, 2]. Venous reflux and its impact on organ dysfunction are generally not a consideration and are frequently overlooked.

In a normal individual, the systemic and pulmonary venous circulations have high compliance. Any decrease in venous compliance causes changes in venous reflux status (VRS). Several factors can change venous compliance and cause disordered venous reflux. These may exist alone or synergistically in critically ill patients. Venous reflux disorders are common in cardiac surgery and congestive heart failure patients. Eljaiek et al. reported that 18.3% of patients who undergo cardiac surgery have a portal pulsatility fraction greater than 50%, which is associated with an increased risk of major complications [3]. In another study, up to 67% of patients had impaired venous reflux [4]. Kidney VRS is a predictor of adverse outcomes in critically ill patients [5, 6]. Venous reflux disorders are associated with a high rate of mortality and persistent renal injury in intensive care unit (ICU) patients [7]. Accurate quantification of VRS is challenging and early symptoms are frequently overlooked [8]. Continuous, dynamic, and quantitative methods are needed for real-time evaluation of VRS at the organ level.

This paper provides a review of the relevant literature regarding the evaluation of VRS in critically ill patients. The search encompassed four databases (Scopus, PubMed, MEDLINE, and Web of Science) to identify peer-reviewed journal articles published from 2000 to October 2022. The databases were queried using the following keywords: (“venous reflux” OR “venous return” OR “venous congestion” OR “venous status” OR “venous excess”) AND (“ultrasonography” OR “ultrasound” OR “critical care ultrasonography” OR “Doppler” OR “point of care ultrasound” OR “central venous pressure” OR “POCUS”). Full-length articles published in English were selected. No conference abstracts, books, editorials, or letters to the editor were selected. Studies involving pregnant women or children were excluded.

This review summarizes the ultrasonographic assessment of venous reflux at the bedside, clarifies the role and clinical significance of VRS in critically ill patients, and establishes a standardized process for bedside ultrasonographic evaluation of VRS.

## 1. VRS in ICU patients

The right ventricular (RV) function is the key to ensuring proper systematic venous reflux. Various diseases[9], including massive pulmonary embolism, inappropriate mechanical ventilation, sepsis, volume overload, ischemia, acute respiratory distress syndrome, drug poisoning, cardiomyopathy, and direct surgical injury, can lead to right ventricular dysfunction (RVD) [10]. Impairment in systemic venous reflux and systemic circulation congestion are significant manifestations of RVD. Among critically ill patients, Zhang et al. [11] discovered that hepatic VRS correlated with RV function. Cirrhosis, venous thrombosis or malformation, abdominal hypertension, and pericardial tamponade are additional causes of venous reflux disorders. A decreased arteriovenous gradient in vital organs, combined with abnormal venous reflux, can impede adequate perfusion. This phenomenon can be exacerbated by the development of interstitial edema following elevated endothelial barrier damage and capillary hydrostatic pressure, which increases the diffusion distance and reduces microcirculation perfusion, ultimately resulting in irreversible damage to organ function [12]. Impaired venous reflux ﻿has been associated with adverse consequences in acute and chronic settings [3, 13-15]. Treatment aiming to normalize VRS may improve the outcome of acute kidney injury (AKI) [16, 17]. The impact of VRS-guided treatment has, however, not been demonstrated in randomized controlled trials.

## 2. Methods of VRS evaluation

### 2.1 Routine methods

The routine methods for evaluating VRS include measuring central venous pressure (CVP) and conducting physical examinations to assess cumulative fluid balance, weight change, and peripheral edema. CVP reflects the local pressure in the vena cava or right atrium and is considered the back pressure of venous reflux. A high CVP is associated with organ dysfunction and poor outcomes among critically ill patients [18]. For every 1mmHg increase in CVP above 7 mmHg, the risk of AKI increases [19]. Venous reflux congestion has been defined as CVP >8 mmHg [20]. However, others believe that the actual threshold is unclear and varies according to physiological state; moreover, the levels of CVP considered deleterious or a trigger for intervention are also unclear [21, 22] . Peripheral edema, cumulative fluid balance and weight change have significant limitations, including lack of objective indicators, inability to evaluate quantitatively, and inconsistent standards.

### 2.2 Bedside evaluation of VRS using Doppler ultrasonography

**Figure 1.**
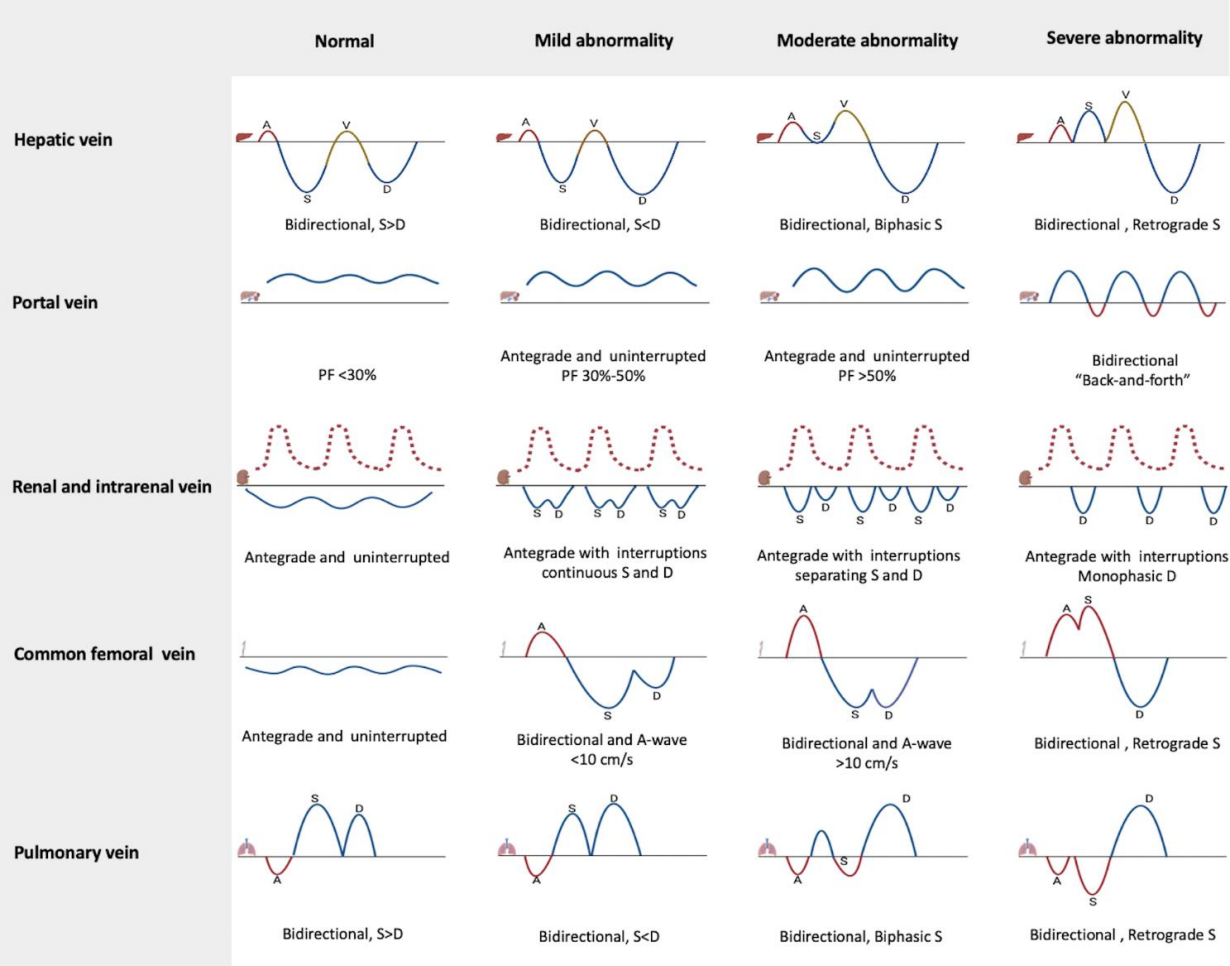
**Grading description of different venous blood flow patterns using pulse wave Doppler**. This figure represents Doppler patterns on hepatic vein, portal vein, renal and intrarenal vein, comom femoral vein and pulmonary vein. Based on the extent of venous load, the flow patterns were classified into four grades: normal, mild abnormality, moderate abnormality, and severe abnormality. Renal and intrarenal Doppler usually capture both an arterial and venous component within the same image. The anterograde, retrograde, and transitional flow of the vein are now represented by blue, red, and brown solid lines, respectively. The anterograde flow of artery is represented by a red dashed line. A-wave: the retrograde wave generated by atrial contraction; S-wave: the antegrade wave resulting from ventricular contraction; V-wave: transitional wave resulting from atrial overfill transition; D-wave: the antegrade wave occurring during ventricular diastole.

Hemodynamic assessment in the ICU increasingly relies on ultrasonography. Doppler ultrasonography is particularly useful for real-time assessment of VRS at the organ level [23]. Bedside ultrasonography enables evaluation of vessel shape and velocity of blood flow. When venous compliance is affected by increased load, the morphology and velocity of the blood flow spectrum will reflect these changes. Normally, venous blood vessels do not expand, and their walls have low tension. Periodic cardiac pressure waves are quickly dampened by the vessel wall and do not travel far. Kerr and Warren discovered that cardiac fluctuations can propagate peripherally in 1925 [24]. With increases in venous load, cardiac pressure waves travel farther, causing corresponding changes in the venous blood flow spectrum known as the “ripple phenomenon.” Venous Doppler assessment can be employed to identify changes that indicate reduced systemic venous compliance [25]. The spectrum of venous blood flow will change accordingly with the severity of venous reflux disorder, whether in acute or chronic venous overload. Ultrasonic waveform morphology of the large systemic and pulmonary veins has been suggested as an indicator of increased venous load [25]. We classified these indicators into four grades based on the extent of venous overload: grade I, normal; grade II, mild abnormality; grade III, moderate abnormality and grade IV, severe abnormality. Figure 1 shows the different flow pattern of the hepatic, portal, renal and intrarenal, common femoral, and pulmonary veins corresponding to these four grades.

#### 2.2.1 Hepatic vein Doppler ultrasonography

The hepatic veins comprise three main veins (right, middle, and left) that merge into the inferior vena cava, which drains into the right atrium. The hepatic veins lack valves, resulting in blood flow that mirrors filling of the right atrium during the cardiac cycle and filling of the right ventricle during diastole. Blood flow in the hepatic veins is typically phasic and bidirectional, and antegrade flow is predominant. Changes in velocity and flow direction may indicate modifications in right atrial pressure (RAP) [26].

Different flow patterns of hepatic veins are shown in Figure 1. On Doppler ultrasonography, the normal hepatic vein waveform exhibits four components: the retrograde A-wave (generated by atrial contraction), antegrade S-wave (resulting from ventricular contraction), transitional V-wave (produced by an atrial overfill transition), and antegrade D-wave (occurring during ventricular diastole). The A-waves are generated by retrograde blood flow in the hepatic veins during atrial contraction when the RAP exceeds the mean systemic filling pressure. The antegrade S-wave is generated by the downward motion of the tricuspid annulus during early systole and corresponds to the peak negative pressure. Increased filling of the right atrium occurs after the tricuspid valve opens owing to early equilibration, resulting in an S-D inversion, which indicates a relatively large D-wave. As the degree of venous congestion intensifies or tricuspid valve regurgitation (TVR) becomes severe, a biphasic pattern can occur in which the S-wave disappears or reverses and merges with the A-wave. The depth and inversion of the V-wave at end-systole may increase with worsening congestion and right atrial volume overload.

#### 2.2.2 Portal vein Doppler ultrasonography

The portal vein gathers blood from the abdominal organs, mainly from the gastrointestinal system. The flow in the portal is dependent on the pressure difference between the portal and hepatic veins and lacks the capacity for automatic flow regulation. ﻿ Different flow patterns of portal vein are shown in Figure 1. The normal waveform of the portal vein is typically antegrade and uninterrupted above the baseline, with any variations in this pattern usually being minor. However, changes in the load caused by retrograde blood flow from the hepatic veins during atrial contraction can be transferred to the portal vein through the hepatic sinusoid, resulting in resistance to blood flow. This can cause slight fluctuations in the portal vein. An increase in RAP can influence the portal circulation, resulting in a pulsatile waveform. When there is a continued increase in RAP and severe TVR, the direction of portal venous flow can be retrograde during systole. This can result in pulsations of the upper and lower streams that move back and forth at the baseline. This results in a “back-and-forth” pattern and can be visualized as the “siren sign” of alternating red and blue in color Doppler mode. The degree of pulsatility in the portal vein can be measured using the portal pulsatility fraction (PF), which is expressed as a percentage and calculated using the formula: 100 × [(Vmax - Vmin)/Vmax]. In this formula, Vmax represents the maximum blood velocity observed and Vmin represents the minimum blood velocity recorded during the cardiac cycle [27].

#### 2.2.3 Renal and intrarenal vein Doppler ultrasonography

As the kidneys are encapsulated, the development of interstitial edema within can cause a rapid increase in interstitial pressure and a reduction in renal perfusion. While the renal vein is relatively distant from the right ventricular, elevation of RAP can propagate to the renal vein via the “ripple” phenomenon, which increases interstitial and tubular hydrostatic pressure within the kidney and further reduces renal perfusion pressure, culminating in progression of AKI [28]. Both the renal and intrarenal veins reflect downstream effects of elevated RAP and interstitial edema within the renal capsule. Therefore, an evaluation of renal venous and intrarenal venous reflux is required.

The renal vein is a direct tributary of the inferior vena cava and alterations in RAP can impact renal venous blood flow. Different flow patterns of renal veins are presented in Figure 1. The typical renal venous Doppler waveform manifests as antegrade and uninterrupted flow below baseline and variation is minimal during the cardiac cycle. However, with increased RAP, renal vein compliance decreases, and venous congestion gradually worsens. This causes a reduction in renal blood flow during systole and a pulsatile venous flow waveform, similar to discontinuous biphasic vein flow. As the venous reflux obstruction worsens, the S-wave diminishes, and the D-wave becomes more prominent. Eventually, the S-wave is completely retrograde and merged with the arterial wave, resulting in a monophasic D-wave below the baseline. Such a “hepatic vein (HV)-like” venous flow pattern indicates obstructive renal venous reflux. The intrarenal vein is also affected by the renal interstitial pressure and an increase in interstitial pressure caused by RAP elevation or other factors can lead to a “HV-like” venous reflux waveform.

#### 2.2.4 Common femoral vein (CFV) Doppler ultrasonography

In the thigh, the CFV extends medially toward the common femoral artery and lies below the ilioinguinal ligament. The CFV must be identified and differentiated from its tributaries, including the saphenous vein. Different flow patterns of CFV are presented in Figure 1. A typical Doppler profile of CFV velocity reveals antegrade flow at a velocity of approximately 10 cm/s. During inspiration, CFV velocity decreases or may demonstrate retrograde flow in individuals with spontaneous breathing. There are a number of factors that can affect the CFV Doppler waveform, including decreased RV function, TVR, venous insufficiency, venous obstruction, and pulmonary embolism [29]. Continuous and antegrade Doppler signals without respiratory fluctuations strongly suggest proximal venous obstruction, which may be a result of iliac vein thrombosis [30]. The CFV Doppler waveform may show a bidirectional pattern with retrograde velocity below 10 cm/s in some patients, a bidirectional pattern with retrograde velocity exceeding 10 cm/s may be seen in patients with RVD and significant TVR. With increasing severity of TVR, this bidirectional waveform is characterized by an asynchronous retrograde wave (combining the A-wave and reversed S-wave) followed by an antegrade D-wave [31].

#### 2.2.5 Pulmonary vein Doppler ultrasonography

The pulmonary veins connect the pulmonary circulation to the left atrium. They have no valves and are relatively short and susceptible to left heart pressure. The VRS of the pulmonary veins can rapidly affect the pulmonary microcirculation and pulmonary artery pressure. They are an important site for the development of extravascular lung water.

Different flow patterns of pulmonary vein are presented in Figure 1. Doppler waves in the pulmonary vein are normally composed of three distinct patterns: atrial contraction generates the retrograde A-wave, ventricular contraction generates the antegrade S-wave, and ventricular relaxation produces the antegrade D-wave. The antegrade S-wave can be further divided into two components: a small component during the onset of systole and a larger component during the mid-to-late phase of systole. The S1-wave is primarily attributed to a decrease in atrial pressure, while the S2-wave is mainly influenced by the RV pressure pulse and a drop in the systolic aortic plane. Impaired left ventricular contractility and RV systolic dysfunction can decrease the systolic aortic plane and RV pressure pulse, leading to reduced forward blood flow in the pulmonary veins. Additionally, during the atrial relaxation period, if the decrease in left atrial pressure is insufficient, the S-wave may decrease, resulting in a disturbance in pulmonary venous reflux. Therefore, a reduction in the S/D ratio can be considered an indicative but nonspecific measure of cardiac dysfunction, suggesting an increased risk of cardiovascular events related to impaired ventricular or atrial function. Severe mitral valve regurgitation can cause the S-wave to exhibit bidirectional or retrograde flow patterns.

**Figure 2.**
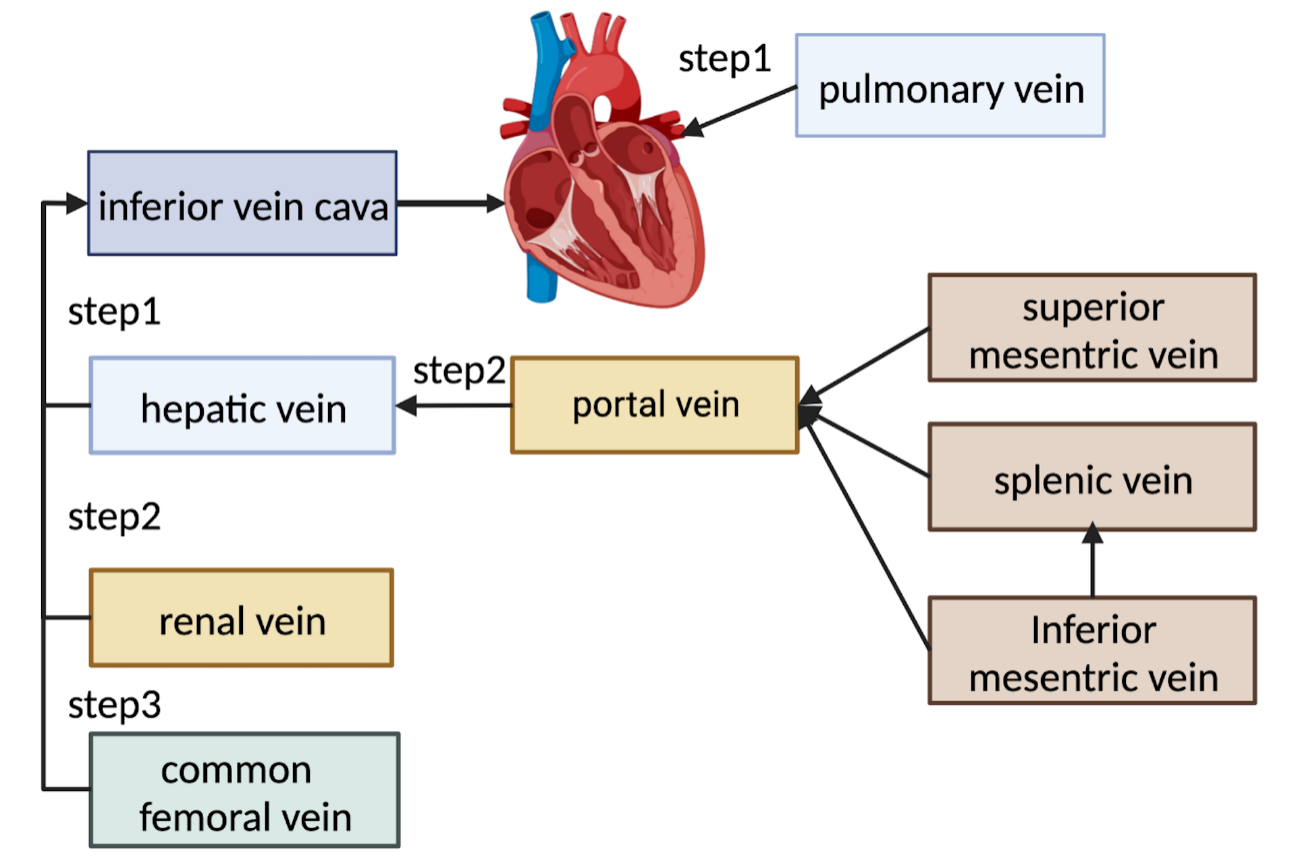
**Assessment of the VRS according to the direction of blood flow**. Step 1: VRS of the hepatic and pulmonary veins. Step 2: VRS of the portal and renal veins. Step 3: VRS of the common femoral vein. VRS: venous reflux status.

## 3. Clinical applications of VRS

During the cardiac cycle, changes in RAP are transmitted to the visceral veins, potentially leading to varying degrees of venous reflux. These reflux patterns can be detected through changes in venous Doppler waveforms [32]. Indicators of abnormal VRS have been linked to poor prognosis in patients with acute and chronic conditions [7]. However, relying on a single index can limit its clinical usefulness. Combining multiple sonographic indicators can enhance the clinical utility of VRS. Several studies have developed scoring systems that integrate these indicators to quantitatively evaluate the degree of venous reflux. One such scoring system is the venous filling ultrasound (VExUS) score, developed by Beaubien-Souligny et al.[4, 10] This score incorporates the diameter of the inferior vena cava and Doppler waveforms of the portal, hepatic, and interlobular renal veins [10, 17, 23]. Elevated VExUS score has been significantly associated with development of AKI in postoperative cardiac surgery patients [33]. The dynamic assessment of VExUS scores during clinical treatment has the potential to reduce the risk of postoperative AKI and improve clinical outcomes.

**Table 1:** Clinical utility of venous reflux status in critically ill patients.

Clinical utility	Summarized point		
**Indicator Reflecting RVD**	Initially, the flow pattern of the HV changes and manifests as “S-wave<D-wave” As RVD worsens, the flow pattern of the HV gradually changes as follows: biphasic S-wave➝retrograde S-wave; and the flow pattern of the PV, RV, and CFV gradually change owing to the “ripple phenomenon”, manifested as a high portal PF (≥50%), “HV-like” renal venous flow pattern, and bidirectional pattern of CFV Application with the direction of blood flow (HV➝PV and RV➝CFV)		
***Indicator for fluid tolerance and guiding fluid therapy***	An abnormal flow pattern (S-wave<D-wave, biphasic S-wave, and retrograde S-wave) of the hepatic and pulmonary veins indicates that the systemic and pulmonary circulations are intolerant to fluids, respectively A normal pattern (S-wave>D-wave) of the HV and a ΔMHV D/MHV D at baseline × 100% by ≥21% indicates a lack of fluid responsiveness		
**Effects on organ function**	Tolerance to VRS disorder differs by organ		
			Liver: Impaired hepatic VRS can cause congestive liver disease
	**Impaired hepatic VRS**		Gastrointestinal: PF is an effective index for evaluation of gastrointestinal reflux, reflect gastrointestinal congestion
	(+) Systemic	(-) Local	
	(+) Systemic	(-) Local	Renal: “HV-like” of renal venous flow pattern suggest renal congestion and evaluated interstitial pressure

RVD, right ventricular dysfunction; HV, hepatic vein; PV, portal vein; RV, renal and intrarenal veins; CFV, common femoral vein; PF, pulsatility fraction; VRS, venous reflux status; MHV D, middle hepatic venous diastolic negative wave S-wave, the antegrade wave resulting from ventricular contraction, D-wave, the antegrade wave occurring during ventricular diastole.

### 3.1 Applications of VRS with direction of blood flow

The VExUS system quantifies VRS but fails to reflect the intrinsic pathophysiological mechanism of venous reflux disorder. Venous reflux follows the order of RV pressure transmission and has a stepwise impact on peripheral veins, indicating that the simple addition of different indicators is not sufficient. Owing to the different distances from the heart and different organs, the alterations and clinical significance of the various venous spectra in pathological conditions are also inconsistent. Among the systemic veins, the hepatic veins, which have no valves and are located closest to the heart, are the most susceptible to venous reflux disorders. Changes in right ventricular load have the earliest effect on the blood flow spectra (morphology and velocity) in the hepatic veins. In clinical practice, the evaluation of systemic VRS should start at the hepatic veins, and then reflux from the portal and renal veins to the CFV should be assessed (Fig. 2). If hepatic vein reflux is normal, but more distal veins such as the portal and renal veins exhibit abnormal retrograde flow, local factors causing reflux obstruction should be considered. Further studies are needed to evaluate the impact of VRS and the direction of blood flow on clinical intervention and prognosis.

## 4. Clinical utility of VRS in critically ill patients

The use of bedside Doppler ultrasonography to measure VRS may be an effective way to detect RVD, assess fluid response or tolerance, and evaluate organ backward load. Although several sonographic indicators have been proposed, their clinical usefulness is hampered when used in isolation or random combinations. To make these indicators easier to understand, we summarize the clinical role of different combinations in Table 1.

### 4.1 VRS is an important indicator of RVD

Continuous, dynamic, and quantitative evaluation of VRS can assess RVD [10, 34]. ﻿Rapid detection of RVD is challenging. Physiologically, RVD is characterized by RV dilation and impaired systemic venous reflux, even without a decrease in cardiac output. While some studies have proposed that CVP >8 mm Hg is a sign of a systemic venous reflux disorder, the clinical usefulness of a CVP threshold value has been controversial because it is susceptible to many factors. Direct evaluation of VRS can more accurately determine the presence of RVD.

**Figure 3.**
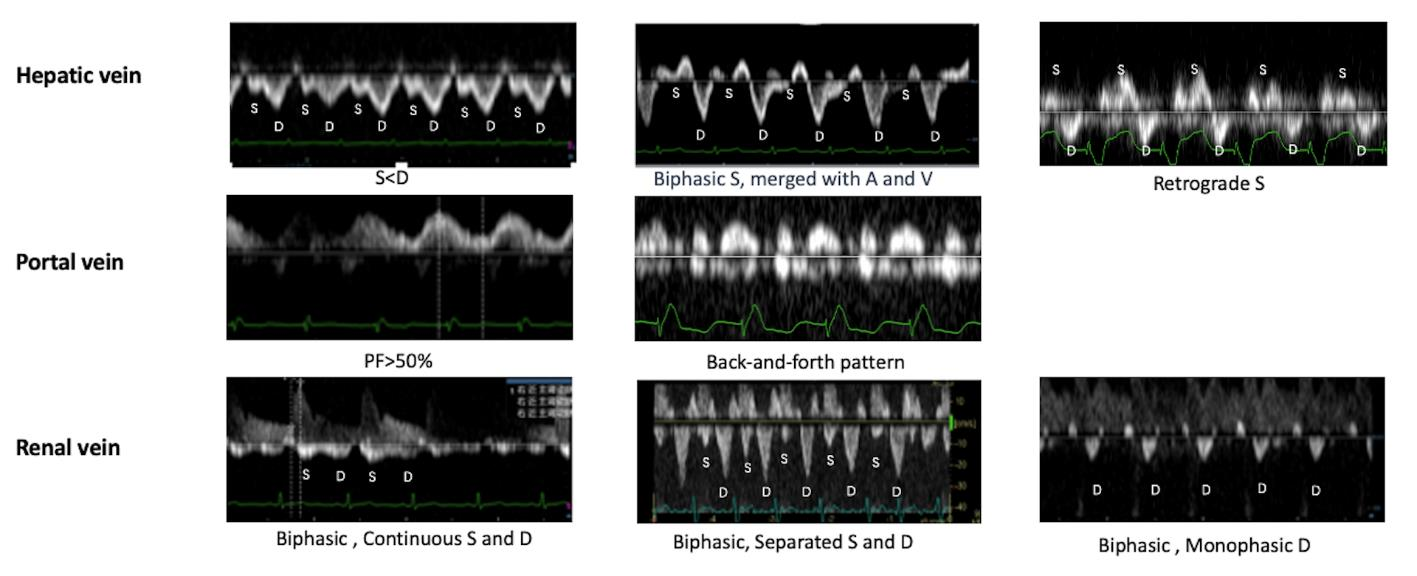
**Abnormal systemic VRS in patients with RVD**. This figure represents Doppler patterns on hepatic vein, portal vein, renal vein in patients with RVD, and the severity gradually increased from left to right. The changes of the hepatic vein are manifested as:S-wave<D-wave, biphasic S-wave (mergerance with A-wave and V-wave) and retrograde S-wave. The changes of the portal vein are manifested as high portal pulsatility fraction, including PF>50% and back-and-forth pattern. The changes of the renal and intrarenal vein are manifested as “HV-like” flow pattern, including biphasic with continuous S-wave and D-wave, biphasic with separated S-wave and D-wave, biphasic with monophasic D-wave; RVD, right ventricular dysfunction.

RVD leads to venous distension in the abdomen and pelvis, resulting in a decrease in vessel capacitance and transmission of the venous pulse distally [35], abnormal changes of different veins are presented in Figure 3. Patients with RVD may show changes in the flow pattern of the hepatic veins, indicating the presence of systemic venous reflux disorders. These changes are characterized by a smaller S-wave amplitude compared with the D-wave amplitude in the Doppler waveform. Furthermore, the subsequent S-wave was bidirectional and merged with A-wave and V-wave, followed by the retrograde S-wave, served as early indicators of disordered systemic venous reflux. Patients with cardiac insufficiency who exhibit hepatic venous waveforms showing an inverted V-wave pattern have higher levels of b-type natriuretic peptide, increased CVP, and a higher incidence of cardiac events [36]. As the venous reflux disorder worsens, there are corresponding changes in the portal vein blood flow spectrum. Portal pulsatility is an ultrasonographic marker of cardiogenic portal hypertension, which is associated with elevated RAP, a hemodynamic indicator of low systemic perfusion [3, 37, 38]. A portal pulsatility fraction ≥50% has been significantly associated with higher positive fluid balance and RVD during cardiac surgery and is an independent predictor of major complications [3]. Changes in renal vein Doppler waveform from a continuous pattern to a “HV-like” pattern have been associated with higher incidence rates of patient death and unplanned hospital admissions [13, 39]. Husain-Syed et al. developed the renal venous stasis index, which calculates the proportion of cardiac cycles without venous outflow [40]. This index has been suggested as a comprehensive Doppler index to assess renal venous congestion, providing a more comprehensive reflection of the complete continuum of renal congestion, and determining the likelihood of developing RVD [41]. ﻿Several reports have primarily linked RVD to the femoral venous Doppler profile. Denault et al. [29] suggested that, on the basis of changes in the frequency spectrum of the femoral vein caused by the “ripple phenomenon” after venous congestion, the status of femoral venous reflux predicts RVD at an early stage.

To summarize, regardless of the underlying cause of RVD, venous reflux disorders often manifest early. The hepatic veins are typically the first to be affected, and as RVD worsens, the blood flow spectra of the portal, renal, and femoral veins change gradually because of the “ripple phenomenon”. It is important to evaluate VRS early to identify RVD, which is associated with poor prognosis [27]. Because of the right ventricular’s unique anatomical structure and physiological function, its function can rapidly decompensate and initiate a vicious cycle. Changes in the reflux spectrum of large systemic veins (hepatic, portal, renal, and femoral) can be used for the early identification and determination of severity, allowing for appropriate treatment.

### 4.2 VRS as an indicator of fluid tolerance to guide fluid therapy

Fluid resuscitation aims to improve organ perfusion and volume responsiveness is frequently the starting point. The goal is to achieve a non-responsive state as the actual endpoint [42]. When fluid exceeds the capacity of the venous system, venous hypertension may result, which can affect venous reflux and cause organ congestion and corresponding dysfunction [43]. Therefore, volume tolerance can be evaluated using VRS [44]. Patients with volume responsiveness may not necessarily have volume tolerance. The goal of resuscitation is to improve organ perfusion, and not to exhaust fluid responsiveness. Various nonmodifiable factors such as age, sex, and underlying conditions can affect an organ’s capacity to tolerate volume. Factors related to injury and intervention, such as inflammation, damage to the endothelium or polysaccharide coating, and the degree of initial fluid resuscitation, can also affect volume tolerance. Early assessment of volume tolerance status can help to guide clinical interventions and prevent volume intolerance. Changes in the venous Doppler spectrum occur earlier than changes in venous circulatory strain [30]. VRS can provide early warning signals before obvious organ dysfunction related to volume intolerance occurs. These signals indicate the different degrees of the adverse and harmful effects of fluid resuscitation and provide a window of opportunity for clinicians to develop more reasonable resuscitation strategies.

In any setting of hypoxia, clinicians should consider whether there is pulmonary circulation intolerance to volume. Pulmonary artery wedge pressure is used to evaluate whether there is an increase in pulmonary capillary hydrostatic pressure. However, measuring wedge pressure requires an invasive procedure and is not routinely performed in clinical practice. Multiple factors affect bedside ultrasonographic assessment of left ventricular filling pressure. The pulmonary vein serves as the interface between the pulmonary circulation and the left atrium. Pulmonary VRS can rapidly influence pulmonary artery pressure and pulmonary microcirculation, which plays a key role in the development of pulmonary edema. Regardless of the cause, elevated left atrial pressure results in a lower pressure difference and a lower volume of blood entering the left atrium through the pulmonary veins. This increases pulmonary vein pressure and contributes to pulmonary edema. Pulmonary VRS can aid in assessing the tolerance of pulmonary circulation to volume expansion. Buffle et al. found that the ratio of peak flow velocity during systole to diastole in the pulmonary vein is the most reliable predictor of readmission in heart failure patients [45].

**Figure 4.**
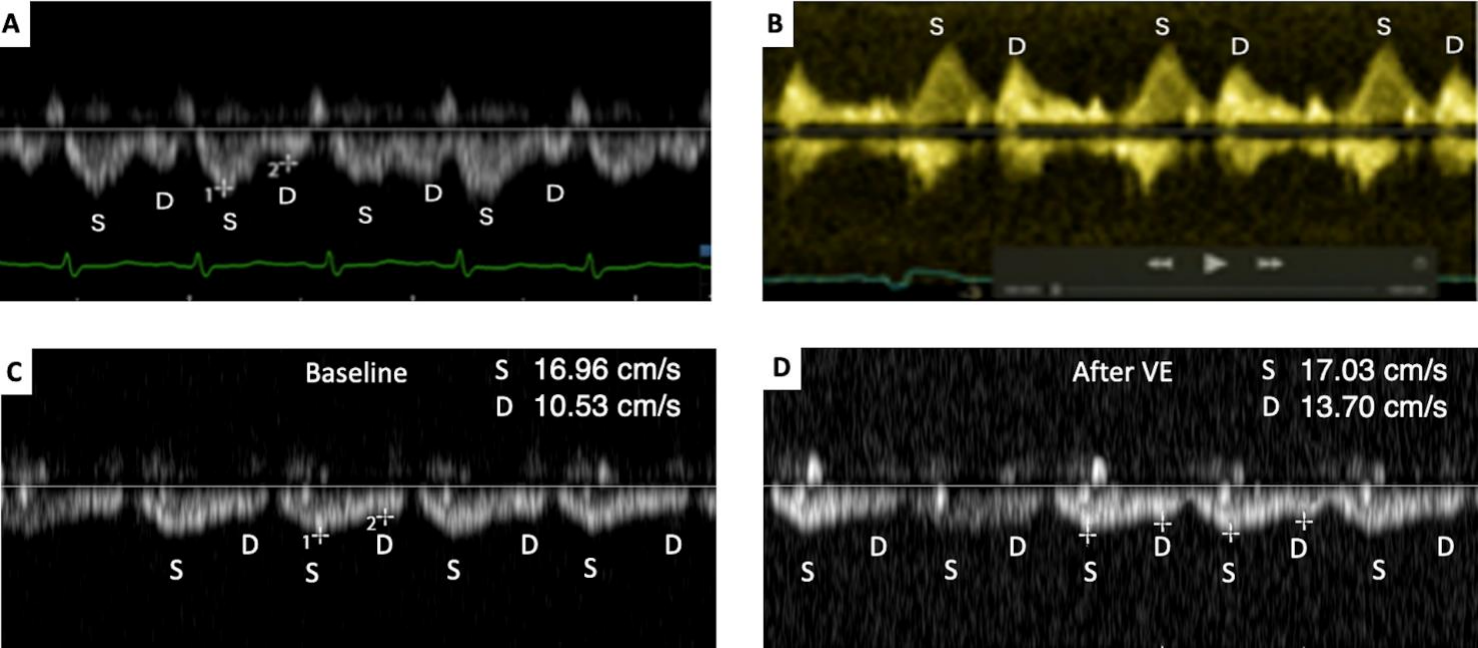
**“Two key indicators” for fluid tolerance and guiding fluid therapy**. (**A**) Normal flow pattern of the hepatic veins indicates fluid tolerance to the systematic circulation. (**B**) Normal flow pattern of the pulmonary vein indicates fluid tolerance to the pulmonary circulation. (**C**) The hepatic vein waveform obtained in baseline. (**D**) The hepatic vein waveform obtained after VE. The D-wave increased with VE, and the ΔMHV D, calculated as (13.7-10.53)/10.53 × 100% = 30.1% (≥21%) indicates a lack of fluid responsiveness. MHV D, middle hepatic venous diastolic negative wave; VE, volume expansion.

According to Hatle et al., increases in left ventricular filling pressure and mean left ventricular pressure are the primary factors contributing to reducing venous flow in the pulmonary veins[46]. In systemic circulation, the hepatic veins are in close proximity to the heart, making them highly responsive to changes in RAP. Abnormal hepatic venous reflux is the initial manifestation of fluid overload. Several studies have demonstrated that assessing the maximum velocity of the S- and D-waves in the hepatic veins can aid in fluid management decisions [47]. Changes in the hepatic veins static D-wave during resuscitation of shock are related to volume responsiveness. An increase of ≥21% in the change of middle hepatic vein S-wave velocity suggests inadequate fluid responsiveness and assists in deciding when to discontinue volume resuscitation [48]. The pulmonary and hepatic veins are most affected by increased left and RAP, making them crucial for evaluating volume tolerance in the clinical setting. These veins serve as “two key indicators” that can indicate intolerance of the systemic and pulmonary circulations to fluid and enable early detection of problems. Changes in the spectra of the hepatic and pulmonary veins can guide fluid therapy and be used to assess safety in a timely manner (Fig. 4).

### 4.3 The effects of VRS on organ function: the role of backward load

Organ perfusion depends on the arteriovenous pressure gradient within the organ [49] and organs have varying degrees of tolerance to increases in venous pressure. The venous reflux of the brain is influenced primarily by intracranial pressure owing to the presence of venous sinuses. Data regarding the effects of impaired VR in the brain are scarce. Visceral organs that have poor autoregulation, such as the liver and gastrointestinal tract, are more affected by increased venous outflow pressure. Experimental studies have demonstrated that acute venous hypertension can cause decreased blood flow in organs including the stomach, small intestine, and colon [3]. Although the kidneys have strong autoregulatory mechanisms, they have two separate capillary networks: cortical perfusion is more influenced by arterial pressure while medullary perfusion is more affected by venous pressure [50]. Evaluating VRS of different organs has always been a challenging issue in clinical practice. By observing the Doppler waveforms of the hepatic, portal, and renal veins, clinicians can determine whether significant venous congestion is present in different organs. Abdominal organ congestion caused by systemic factors first manifests as a change in hepatic VRS. Therefore, it is important to evaluate hepatic venous blood flow first; if unchanged, then venous congestion should be adequately evaluated in other organs to search for locally relevant factors.

The hepatic veins, lacking valves, are highly susceptible to systemic congestion caused by elevated RV pressure. Throughout the cardiac cycle, they reflect blood flow from right atrial filling and RV filling during diastole[40]. Impaired hepatic venous reflux can result in congestive liver disease [51]. Passive congestion leads to hepatic sinusoidal hypertension, which has direct effects on liver biochemistry [52]. Consequently, these liver function effects can have long-lasting implications that persist for many years [53].

The portal vein blood flow spectrum is an effective indicator of gastrointestinal venous reflux. In the gastrointestinal tract, increased venous pressure causes relative ischemia of the intestinal villi, which causes ischemic injury and acidosis at the tip of villous cells and affects sodium and phosphate transport metabolism. Relative ischemia in the intestine can also cause disordered function of the intestinal barrier, which may alter the gut microbiota environment and allow pathogenic microorganisms and bacterial endotoxins to enter the bloodstream [54]. Portal vein pulsations serve as an ultrasonographic indicator of portal hypertension related to cardiac dysfunction, allowing for the identification of significant gastrointestinal congestion in clinical practice [27] . These pulsations are associated with elevated RAP, low systemic perfusion hemodynamic markers, and worsening New York Heart Association functional classification. Notably, Styczynski et al. reported that a portal flow rate of 50% or higher is the most reliable predictor of elevated serum bilirubin levels in patients with heart failure, suggesting that venous congestion can contribute to liver dysfunction [37]. Thus, portal vein Doppler examination holds potential as an assessment tool for evaluating the interplay between the cardiac and gastrointestinal system [30]. It is important to acknowledge that portal vein blood flow serves as an indicator of venous congestion, and it is crucial to rule out other causes of portal hypertension, such as cirrhosis and portal vein thrombosis.

The kidney has two microcirculation systems, one for the glomeruli and one for the renal tubules. Changes in intraperitoneal pressure, venous reflux pressure, and interstitial edema can affect renal interstitial pressure, which has an impact on the microcirculation. “HV-like” changes in the renal venous spectrum indicate that reflux is obstructed, which influences perfusion of the renal medulla. Impaired renal venous reflux can initiate a detrimental cycle involving renal insufficiency, heightened intra-abdominal pressure, neurohormonal activation, fluid overload, and diuretic resistance [55]. Consequently, this cascade leads to further elevation of RAP [28]. The elevated RAP is transmitted to the renal vein, resulting in increased interstitial and tubular hydrostatic pressure within the enclosed kidney. This increases endothelial leakage and further reduces renal perfusion pressure, leading to progression of AKI in critically ill patients [28]. Spiegel et al. [7] reported that abnormal hepatic and portal venous flow spectra significantly heighten the likelihood of renal adverse events in critically ill patients.

### 4.4 Challenges and limitations of VRS evaluation in ICU patients

Despite the benefits of ultrasonographic Doppler assessment of VRS, challenges and limitations exist. First, the interpretation of ultrasound imaging in critically ill patients can be challenging. For example, the hepatic vein waveform can be influenced by TVR [56] and liver cirrhosis with fibrosis [57]. Additionally, pulsatile portal flow has been reported in healthy exercising volunteers [58]. Other causes of portal hypertension, such as cirrhosis and portal vein thrombosis, must be excluded. Arrhythmias are also common causes that affect the Doppler waveform in venous ultrasonography. Furthermore, the assessment of VRS should be based on the direction of blood flow. The combination of different venous Doppler images may indicate different clinical scenarios. Although the VRS is easily evaluated and its evaluation is cost effective in the ICU, ultrasonography is operator dependent, and operators must be adequately trained. It is important to note that the accuracy of VRS assessment relies on the operator's expertise, such as the ability to identify the impact of arrhythmias on the images. A training curriculum and methods to assess the competence of operators are imperative. Moreover, the venous reflux waveform primarily demonstrates morphological alterations, and the determination of quantitative cutoff values remains unclear. The absence of standardized therapeutic protocols based on VRS poses challenges in studying its impact on patient outcomes.

#### Conclusion

The use of bedside Doppler ultrasonography to measure VRS may be effective for evaluating organ perfusion, assessing the hemodynamic response to fluid administration [59, 60], detecting RVD, and predicting outcomes in critically ill patients, including occurrence of AKI [15, 29], length of hospital stay, and mortality [7]. Bedside ultrasonography can guide treatment and be used to monitor the effects of diuretic therapy [61]. Although case reports have suggested the potential benefits of dynamically monitoring venous reflux disorders to improve patient outcomes, there is a scarcity of prospective randomized controlled studies in this field. Future research should prioritize the development of efficient and precise evaluation protocols, along with the implementation of effective training programs. Furthermore, prospective randomized controlled studies are required to validate the clinical applications of VRS in various clinical contexts, taking into account the direction of blood flow. Finally, evaluating whether VRS-guided treatment can enhance outcomes in critically ill patients warrants investigation.
